# Diplomas in Radiography

**Published:** 1921-09-03

**Authors:** 


### The Editor's Letter-Box.

**Correspondence on all subjects is invited, but we cannot in any way be responsible for the opinions expressed.**

### Diplomas in Radiography.

*To the Editor of* The Hospital.

Sir,—I have been following with much interest the correspondence in your columns concerning the new examination for radiographers; also the article on radiography in a recent issue.

Having been one of the first to obtain the radiography certificate granted by Guy's Hospital, after taking an eight months' course, which seems to more than cover the subjects required by the Society of Radiographers, and since then having worked under two well-known radiographers, I am amazed to find that all this tuition and experience will count as nothing unless I waste time and money in sitting an examination of certainly no higher standard than that I have already passed.

I fully agree that radiographers' assistants should have a settled status and that a universal examination such as that inaugurated by the Society will be immensely to their benefit. But surely those who have had the advantage of an eight months' training in a hospital giving such opportunities for experience in practical work and tuition by experts as Guy's Hospital should be accepted as trained radiographers without further examination.

This matter does not so seriously affect such of us whose work is in London, but many of those who trained with and after me now hold posts in the Colonies, and it is on their behalf I add my protest to those of "Spark Gap" and "E. D. H." B. G.

